# Mechanical and Functional Properties of a Novel Apatite-Ionomer Cement for Prevention and Remineralization of Dental Caries

**DOI:** 10.3390/ma12233998

**Published:** 2019-12-02

**Authors:** Rie Imataki, Yukari Shinonaga, Takako Nishimura, Yoko Abe, Kenji Arita

**Affiliations:** 1 Graduate School of Dentistry (Department of Pediatric Dentistry), Osaka Dental University, 8-1, Kuzuhahanazono-cho, Hirakata-shi, Osaka 573-1121, Japan; imataki-r@cc.osaka-dent.ac.jp; 2 Department of Pediatric Dentistry, School of Dentistry, Osaka Dental University, 8-1, Kuzuhahanazono-cho, Hirakata-shi, Osaka 573-1121, Japan; nisimura@cc.osaka-dent.ac.jp (T.N.); abe-y@cc.osaka-dent.ac.jp (Y.A.); arita-k@cc.osaka-dent.ac.jp (K.A.)

**Keywords:** glass-ionomer cement, hydroxyapatite, reinforcement, mechanical property, functional property

## Abstract

Especially in pediatric dentistry, prevention by the control of initial lesions prior to cavitation is very important, and application of a pit and fissure sealant is essential to achieve this. Numerous reports have suggested that resin-based sealants are inferior to sealants based on glass-ionomer cement (GIC), because of GIC’s many advantages, such as fluoride ion release properties and its good adhesion to tooth structures. However, the use of GIC is impeded due to its low flexural strength and fracture toughness. In this paper, we developed and characterized an apatite-ionomer cement (AIC) that incorporates hydroxyapatite (HAp) into the GIC; this development was aimed at not only reinforcing the flexural and compressive strength but also improving some functional properties for the creation of the material suitable for sealant. We examined the influence of differences in the compounding conditions of GIC powder, liquid, and HAp on flexural and compressive strengths, fracture toughness, fluoride ion release property, shear bond strength to bovine enamel, surface pH of setting cements, and acid buffer capability. These methods were aimed at elucidating the reaction mechanism of porous spherical-shaped HAp (HApS) in AIC. The following observations were deduced. (1) HAp can improve the mechanical strengths of AIC by strengthening the cement matrix. (2) The functional properties of AIC, such as acid buffer capability, improved by increasing the releasing amounts of various ions including fluoride ions. The novel AIC developed in this study is a clinically effective dental material for prevention and remineralization of tooth and initial carious lesion.

## 1. Introduction

The plaque biofilm composition alters to a less cariogenic flora if a caries lesion is isolated by biofilming the oral environment [1]. This understanding of dental caries has led to the use of minimal invasive (MI) management, which reformed the policy issued by FDI World Dental Federation in 2016 [2]. This management advocated biological approach policies such as the remineralization of demineralized enamel and dentin; further, it encouraged minimally invasive operative interventions to ensure tooth survival and ignored the traditional surgical approach [3]. In recent years, dentists have realized that enamel and dentin are irreplaceable and that the preservation of as much tooth substance as possible is imperative; this implies not only the prevention of compromising any tooth strength but also the maintenance of the dental pulp’s health [4]. Therefore, dental materials that can adhere to the tooth structure and promote remineralization are essential for MI management.

Additionally, early childhood caries (ECC) is defined as the presence of one or more decayed (non-cavitated or cavitated lesions), missing, or filled (due to dental caries) surfaces in any primary tooth of a child under six years of age. Currently, ECC affects more than 600 million children worldwide, and remains largely untreated [5]. Dental caries is preventable because the pathophysiological processes of dental caries cause mineral loss, which occurs as a result of an imbalance between the demineralization and remineralization of tooth structure. Therefore, the Bangkok declaration for ECC by the International Association for Paediatric Dentistry (IAPD) [5] proposed three phases for ECC prevention: (i) primary prevention by improving the oral health literacy of parents/caregivers and healthcare workers, limiting children’s consumption of sugar in drinks and foods, and daily exposure to fluorides; (ii) secondary prevention by the control of initial lesions prior to cavitation that may include more frequent fluoride varnish applications and applying pit and fissure sealants to susceptible molars; and (iii) tertiary prevention by the arrest of cavitated lesions and tooth-preserving operative care.

Adhesive dentistry originated with the work of Buonocore in 1955 [6], who demonstrated that the treatment of enamel with phosphoric acid resulted in a porous surface, which could be infiltrated by resin to produce a strong micromechanical bond. However, the material exhibiting chemical bonding with tooth structure was developed by Smith in 1968 [7], and it resulted in the introduction of polycarboxylate cement. Further work by Wilson and Kent [8] resulted in the introduction of the conventional glass-ionomer cement (GIC), called glass polyalkenoate cement initially. Polycarboxylate cements are now seldom used, as GIC has a wider range of applications and is easier to use [9]. For the prevention in the case of primary and permanent teeth, pit and fissure sealants were recommended, as described above. Specifically, until erupting teeth reach full occlusion, pits and fissures will form a favorable environment for plaque accumulation and bacterial growth. It was also reported that pits and fissures in molars, which comprise only 3% of the total tooth surface area, exhibit about 70% of the caries or fillings in children [10]. In general, composite resin or GIC are used as sealant materials. Resin-based sealants are difficult to apply during erupting teeth, when it is the period of the highest caries risk, because resin materials require strict moisture control. Resin-based sealants undergo failures ranging from 5 to 10% each year because they are technique-sensitive materials [11]. Any partial loss or defects of resin-based sealants leaves a highly caries-susceptible surface unprotected. Beiruti et al. reported that the GIC sealant appeared to have four times higher chance of preventing caries development in re-exposed pits and fissures of occlusal surfaces in first molars than composite resin sealant material over a one- to three-year period [12]. Moreover, GICs exhibit many attractive features such as adhesion to tooth structure, good biocompatibility, and fluoride ion release, which results in the increased resistance of fissure demineralization [13,14]. Therefore, GICs might be a suitable alternative to composite resin sealants as they are less sensitive to moisture [13]. In particular, for children, people with special needs, and those with dental fear, composite resin sealant or filling that require strict moisture protection are often unsuitable [15]. However, the use of GIC has been limited due to its poor mechanical properties, including low flexural strength and fracture toughness compared to resin-modified GIC and composite resin [16,17,18]. 

In this paper, we report the development of a novel material that is termed as apatite-ionomer cement (AIC). This material is a porous and spherical-shaped hydroxyapatite (HApS), having less than 1/600 the strength of GIC glass particle, incorporated into conventional GIC. We suggest that HApS not only reinforced flexural and compressive strength, but also enhanced fluoride and the other ion release properties as well as the antibacterial property against cariogenic bacteria of conventional GIC [19,20,21,22,23]. However, the shear bond strength, fracture toughness, solubility, and acid buffer capability of AIC with HApS have not been examined; moreover, the reaction mechanism that led to the aforementioned effects by HApS has not yet been elucidated. Therefore, we attempted in this study to clarify the role and mechanism of HApS in AIC by examining properties such as shear bond strength, fracture toughness, solubility, pH change of cement surface, and acid buffer capability. The tested null hypotheses of our study are as follows: (I) no difference is found between the mechanical strength of GIC and AIC and (II) no difference is found between the functional property of GIC and AIC.

## 2. Materials and Methods 

### 2.1. Sample Preparation

Commercial conventional GICs meant as pit and fissure sealants were used as two control groups (GIC groups). The base material of the two AIC groups in this study was Fuji III (GC Co., Tokyo, Japan; powders’ lot nos. 1903151 and 1810011, liquids’ lot nos. 1904011 and 1809011) with the following formation: the powder contained fluoroaluminosilicate glass; the liquid contained polyacrylic acid, polybasic carboxylic acid, and water. The powder of the AIC groups was added with spherical-shaped HAp powder having an average particle size of 20 μm (HApS; Taihei Chemical Industrial Co., Ltd., Osaka, Japan) and Fuji III-powder before mixing with Fuji III-liquid.

In this study, we established a group that maintained a certain amount of Fuji III but had different amounts of Fuji III-powder and HAp; this was aimed to investigate the effect of HAp addition. Each group was prepared according to the composition shown in Table 1. The numbers -0.9 and -1.2 in each group represent the powder/liquid ratio of Fuji III (Fuji III P/L); thus, the groups with the same numbers include the same amount of Fuji III-powder and Fuji III-liquid.

### 2.2. Measurements of Mechanical Strength

#### 2.2.1. Flexural Strength

Beam-shaped samples (*n* = 5) of each group measuring 25 mm × 2 mm × 2 mm for flexural strength tests were prepared in a stainless steel split mold following the procedures outlined in ISO9917-2:2017 [24]. The cement was inserted into the mold with a syringe, covered with a polyester strip at the top and bottom surfaces, and compressed through a glass plate with a load of 500 g for 10 min. The samples were stored at 37 °C with a relative humidity of 100% for 50 min. The samples were then carefully removed from the molds and stored in artificial saliva (Saliveht Aerosol, Teijin Ltd., Osaka, Japan) for a further 23 h at 37 °C, and subjected to a three-point bending test using a universal testing machine (AGS-X, Shimadzu Co., Kyoto, Japan) at a crosshead speed of 0.5 mm/min.

#### 2.2.2. Compressive Strength

Cylindrical specimens (*n* = 5) measuring 4 mm in diameter and 6 mm in height were prepared in a stainless steel split mold for compressive tests, following the procedures outlined in ISO9917-1 [25]. After 24 h of storage in artificial saliva, samples were tested using a universal testing machine (AGS-X, Shimadzu Co., Kyoto, Japan) at a crosshead speed of 1 mm/min.

#### 2.2.3. Fracture Toughness

Fracture toughness was determined according to the method outlined in our previous study for single-edge notch specimens loaded in transverse bending [26]. Knife-edge notch specimens (*n* = 10) having dimensions of 25 mm × 2.5 mm × 5 mm with a 0.5 mm notch width and 2.5 mm depth were prepared in a polyethylene split mold. Similar to the procedure for the flexural strength measurement, the three-point bending test was conducted in a universal testing machine (AGS-X, Shimadzu Co., Kyoto, Japan) at a crosshead speed of 0.5 mm/min. Fracture toughness, *K_IC_* (MPa m^1/2^), was calculated from the following Equation:

*K_Q_* = (*P_Q_S*)/*BW*^3/2^) ⋅ *F*(*a*/*W*), (1)

where *P_Q_* is the peak load (kN), *S* is the span (cm), *B* is the specimen thickness (cm), *W* is the specimen width (cm), and *a* is the crack length (cm). *F*(*a*/*W*) is a function of *a*/*W* and is calculated as follows:

*F*(*a*/*W*) = 3(*a*/*W*)^1/2^ × [1.99 − (*a*/*W*)(1 − *a*/*W*)(1 − *a*/*W*) × (2.25 − 3.93 *a*/*W* + 2.7a^2^/W^2^)/2(1 + 2*a*/*W*)(1 − *a*/*W*)^3/2^. (2)

### 2.3. Measurement of Shear Bond Strength

Samples of bovine incisor teeth were used to measure the shear bond strength of the enamel. The teeth were embedded in a mounting resin (KM-U, PRESI Co., Hargen, Germany) in cylindrical rubber molds, with the labial surface at the bottom of the mold. The labial surface of each tooth was trimmed with a low-speed trimmer and was ground flat on wet SiC paper, up to 1200 grit, until the enamel 0.3 mm below the surface was exposed. A Teflon split mold with a cylindrical hole (3.6 mm in diameter and 2.0 mm in height) was clamped to the prepared enamel surface. The mold was filled with the cements and left at room temperature for 10 min. The samples with molds were stored at 37 °C with a relative humidity of 100% for 50 min. The samples were then carefully removed from the molds and stored in deionized water for a further 23 h at 37 °C. The prepared specimens (*n* = 10) were immersed in artificial saliva in a 37 °C incubator. After 24 h, 1 month, and 3 months, the shear bond strength test was conducted using a universal test machine (AGS-X, Shimadzu Co., Kyoto, Japan) at a crosshead speed of 0.5 mm/min. The experimental protocol was approved by the Committee for Animal Experiments of Osaka Dental University (#19-02010).

### 2.4. Fluoride Ion Release Test

Five cylindrical specimens (10 mm in diameter and 2 mm in thickness) were prepared for each group. The samples were individually suspended by a cotton thread in 18 mL of deionized water in sealed containers and were stored at 37 °C. For the measurements, each disk was removed from the water, washed by immersion in 2 mL of water, dried on filter paper, and immediately immersed in 18 mL of fresh distilled water for further elution. A volume of 2 mL of a total ionic strength adjustment buffer solution (TISAB III, Thermo Fisher Scientific, Beverly, MA, USA) was added to the water sample. The fluoride ion concentration, in ppm, was measured daily for 5 days using a fluoride-selective electrode (6561-10C, Horiba Ltd. Co, Kyoto, Japan) connected to a pH/ion meter (D-53, Horiba Ltd., Kyoto, Japan). The results were evaluated in terms of μg/cm^2^ by calculating the amount of fluoride ions released from the surface of the specimen.

### 2.5. Measurement of Solubility Rate

The cement was inserted into the mold (20 mm in diameter and 1.5 mm in thickness) and covered with a polyester strip at the top and bottom surfaces, compressed with a glass plate under a load of 500 g for 150 s, and then stored at 37 °C with a relative humidity of 100%. After 1 h from initiating the cement mixing, the specimens were remolded. Two specimens were suspended by a nylon thread into 50 mL of distilled water in a conical flask with a glass stopper and stored at 37 °C. After 24 h of storage, the specimens were removed from the conical flask. The solution in the glass bottle was evaporated at 100 °C in an oven for 3 days. The rate of disintegration, D (%), was calculated according to the following Equation:

D = [(A’ − A)/B],(3)

where A is the weight of the glass bottle measured before the test (g), A’ is the weight of the glass bottle measured after the test (g)(A’ − A was hence the weight of the residue after the immersion of the specimens), and B is the weight of two specimens before immersion into the distilled water. 

### 2.6. Measurement of Surface pH of Setting Cements

The cement mix was condensed into the mold that was 10 mm in diameter, covered with a sampling sheet (Y011, Horiba Ltd., Kyoto, Japan), and moistened with 50 μL of deionized water. The pH values of the cement surface at the specified time were measured from 2 min to 12 weeks after the start of mixing using a flat-typed ion-sensitive field effect transistor (ISFET) pH electrode (0040-10D, Horiba Ltd., Kyoto, Japan) connected with a pH/ion meter (D-53, Horiba Ltd., Kyoto, Japan). The specimens were wrapped with moistened filter paper and stored at room temperature until the measurement time to ensure that the specimens did not dry up.

### 2.7. Acid Buffer Capability Test

Specimens that were placed at room temperature for 10 min and stored in distilled water for 1 week were prepared (*n* = 12/each storage time) using cylindrical molds (10 mm in diameter × 2 mm in thickness). A 5 mL portion of lactic acid solution of pH 4.0 was poured into a 50 mL plastic tube, and the specimen was placed into the solution. A pH electrode (6252-10D, Horiba Ltd, Kyoto, Japan.) connected to a pH/ion meter (D-52, Horiba Ltd., Kyoto, Japan) was inserted in the center of the tube. Then, pH measurement was conducted after 30 and 60 min. 

### 2.8. Statistical Analysis

The data were analyzed by *t*-test (KaleidaGraph 4.00, SYNERGY SOFTWARE, Reading, PA, USA) and presented in the form of mean ± standard deviation (SD) with *p*-values < 0.05 being considered statistically significant. The confidence interval was set at the 95% confidence level.

## 3. Results

### 3.1. Mechanical Strength

Table 2 summarizes the mean flexural and compressive strengths as well as the fracture toughness (with the corresponding SDs) obtained for the GIC and AIC groups. Compared between the same Fuji III P/L groups, the flexural strengths, compressive strengths, and fracture toughness of AIC-0.9 and AIC-1.2 were significantly higher than those of GIC-0.9 and GIC-1.2 (*t*-test; *p* < 0.01). Compared between the same total P/L groups, the flexural strength and fracture toughness of AIC-0.9 were significantly higher than those of GIC-1.2; however, there was no significant difference between their compressive strengths.

### 3.2. Shear Bond Strength to Bovine Enamel

As shown in Table 3, there were no significant differences between GICs and AICs in shear bond strength to bovine enamel after 24 h and 1 month. After 3 months, the shear bond strength of AIC-0.9 was significantly higher than that of GIC-0.9 (*p* < 0.05). The shear bond strength values after 1 and 3 months in all groups were significantly higher compared to the value after 24 h (*p* < 0.01). There were no significant differences in the shear bond strengths between the periods after 1 and 3 months in GIC-1.2, AIC-0.9, and AIC-1.2.

### 3.3. Fluoride Ion Release Property and Solubility

Table 4 summarizes the mean cumulated fluoride ion release doses for 5 days and solubility rate for 24 h (with the corresponding SDs) obtained for the GIC and AIC groups. Compared between the same Fuji III P/L groups (GIC-0.9 versus AIC-0.9, and GIC-1.2 versus AIC-1.2), there were no significant differences in the fluoride ion release dose and disintegration rate. However, for the same total P/L groups, the fluoride ion release dose and disintegration rate of AIC-0.9 were significantly higher than those of GIC-1.2.

### 3.4. Surface pH Changes

**Figure 1:**
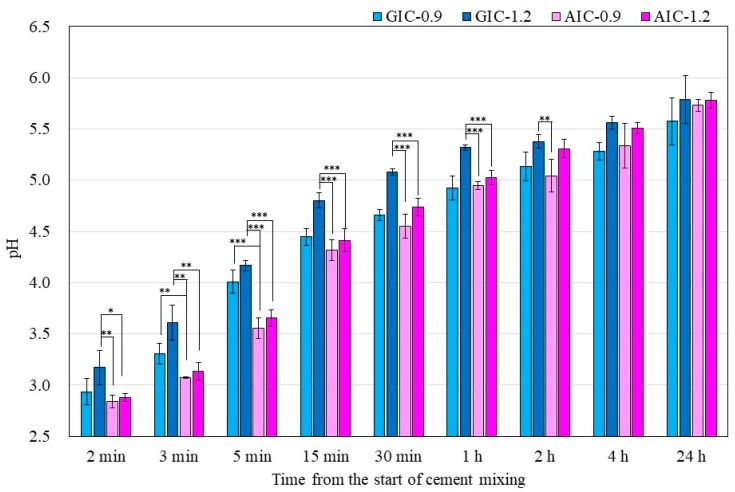
Surface pH of specimens during setting (*t*-test–* *p* < 0.05, ** *p* < 0.01, *** *p* < 0.001).

Figure 1 shows the surface pH values of the specimens during setting from 2 min to 24 h from the start of cement mixing. The surface pH values of AICs tended to be significantly lower than those of GICs. However, there were no significant differences in the surface pH values of GICs and AICs after 4 h. 

**Figure 2:**
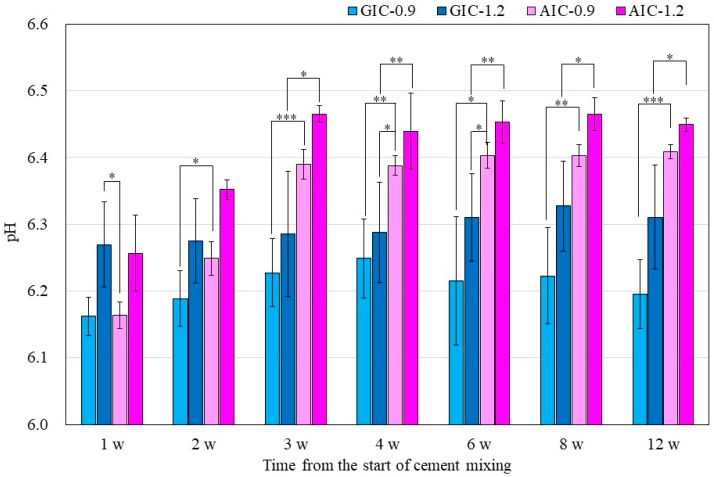
Surface pH of specimens after setting (*t*-test–* *p* < 0.05, ** *p* < 0.01, *** *p* < 0.001). w: week/weeks.

Figure 2 also shows the surface pH values of GICs and AICs stored for 1 week to 12 weeks. After 3 weeks, the surface pH values of AIC-0.9 and AIC-1.2 significantly increased compared to GIC-0.9 and GIC-1.2, respectively.

### 3.5. Acid Buffer Capability

**Figure 3:**
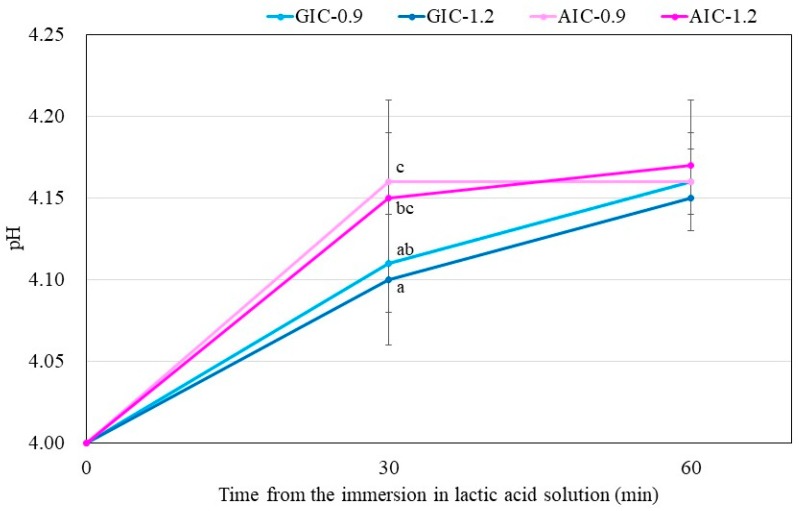
Changes in pH of lactic acid with immersed samples that were stored for one week after the start of cement mixing. Different letters indicate significant differences (*t*-test–*p* < 0.05). There were no significant differences between the values after 60 min immersion.

Figure 3 shows the change in pH of the lactic acid solution when the specimen stored for one week after the start of mixing is immersed in the lactic acid solution. In all groups, there was no significant difference between the pH of the lactic acid solution 30 min after the sample immersion and that after 60 min. Compared between the same Fuji III P/L groups (GIC-0.9 versus AIC-0.9 and GIC-1.2 versus AIC-1.2), there were significant differences in the pH of the aqueous lactic acid solution 30 min after sample immersion. In addition, compared between the same total P/L groups, the pH of the aqueous lactic acid solution 30 min after AIC-0.9 specimen immersion was significantly higher than that after GIC-1.2 specimen immersion.

## 4. Discussion

In the present study, compared between GIC-1.2 and AIC-0.9 with the same total P/L, the flexural strength of AIC-0.9 was significantly higher than that of GIC-1.2, and there was no significant difference between their compressive strengths. Our previous study [20], which used GIC for the restoration (Fuji IX GP Extra (GC Co.)), also reported the same inclinations of these results in the present study. Further, comparing GICs and AICs with the same Fuji III P/L, GIC-0.9 versus AIC-0.9 and GIC-1.2 versus AIC-1.2, the flexural and compressive strengths of AICs were significantly higher than those of GICs. These results showed that porous spherical-shaped HAp can directly enhance mechanical strengths. The porous spherical-shaped HAp particle has extremely low micro-compressive strength (0.06 ± 0.06 MPa [22]) compared to that of Fuji III glass (3.7 ± 0.4 MPa in the preliminary study). GICs are brittle materials with low fracture toughness. Fracture toughness is the measurement of a material’s ability to resist catastrophic failure, and understanding the fracture toughness of AIC is an important parameter [27]. In the present study, the fracture toughness of AIC used for pit and fissure sealant was measured for the first time. In Table 2, the fracture toughness values of AICs were significantly higher than those of GICs with both the same total P/L and Fuji III P/L. Shigeta et al. [28] reported that several concentrations of resin components were added to the conventional restorative GIC, mixed at three different P/Ls. The fracture toughness of the specimens was measured, and an optimum range of additional resin to the liquid of the conventional GIC to increase the fracture toughness was found. Moreover, it was suggested that hydration gel structures were formed and dispersed into the matrix layer of the resin component added to conventional GIC, increasing the specimens’ fracture toughness because the gel structures worked as fillers in the matrix [28]. The mechanical strength of dental cement is affected by the strength of the core and matrix as well as the bond strength between the core and matrix [29]. Thus, we considered that the structural reinforcement of the matrix, such as via the dispersion of HAp nanoparticles in AIC, improve not only the flexural strength but also the fracture toughness. Considering the adhesive property of GIC, Tyas and Burrow mentioned that the basic bonding mechanism was an ionic attraction of the carboxyl groups in the cement with the calcium (Ca^++^) in the apatite of enamel and dentine [9]. Phosphate ions (negatively charged) and Ca^++^ (positively charged) are displaced into the unset cement [9,30]. This results in an intermediate layer between GIC and HAp of tooth, termed as “ion-exchange” [31], “hybrid” [32], and “intermediate” [33] layers. In our previous study [19,20], we observed innumerable nanoparticles dispersed from HApS into the AIC matrix layer using SEM. Consequently, we considered that polyacrylic acid reacted with innumerable HAp nanoparticles and that the innumerable strong intermediate layers, as seen in the adhesive reaction of GIC to enamel and dentin, were formed in the matrix layer of AIC; therefore, AIC exhibited greater mechanical strength compared to that of GIC. Chieruzzi et al. also mentioned that the reaction mechanism between HAp and GIC was similar to the mechanism of adhesion of GIC to enamel and dentine [34].

GIC exhibits adhesion to enamel and dentin. Since the Fuji III used in this study is commonly used in pit and fissure sealant materials, we investigated its adhesion to enamel in the present study. The shear bond strength values of GICs and AICs were not significantly different after 24 h and 1 month of storage; however, the values of AICs tended to be greater than those of GICs after 1 month of storage. Moreover, after 3 months of storage, the shear bond strength values of AIC-0.9 were significantly higher than those of GIC-0.9, and the same aforementioned tendency was observed after 1 month of storage. Lucas et al. [26] reported that the shear bond strength values of Fuji IX GP (GC Co.) and Fuji IX GP with HA 200 (Taihei Co.), with HAp of highly pure hexagonal columnar crystals, to human dentin were not significantly different at any time after 15 min, 1 h, 24 h, 7 days, and 56 days of storage; the values of GIC and GIC with HA 200 were almost the same. Arita et al. [35] suggested that HAp particles with a high specific surface area and low crystallinity may be suitable for incorporation into AIC. It was also considered that the difference in the kinds of HAp affected the mechanical strength, including flexural, compressive, and shear bond strengths. Although there was no difference caused by P/L, there was a tendency for P/L to be larger and the adhesive strength to be higher with less liquid in both GICs and AICs. Hibino et al.’s reports suggested that the bond strength of cements to metal was affected by the P/L [36]; however, no significant increases in the bond strength to bovine enamel or dentine were found in the GIC specimens mixed with different P/Ls [37]. The shear bond strength of GIC to tooth structures is affected by several factors such as surface roughness or the surface condition of the tooth structure [38]. In the present study, no surface treatment was performed, such as using conditioning or acid etching, on the substrate prior to bonding. Conventional GICs bond to enamel, even with the presence of a smear layer; however, surface conditioners have been found to improve the bond strength [38]. Further studies should focus on the surface conditions during the shear bond strength test. Peumans et al. reviewed the clinical efficacy test for 3- and 2-stage etch and rinse adhesives, 2- and 1-stage self-etch adhesives, and glass-ionomer adhesives. Comparing the retention of class-V adhesive restorations to determine the clinical bonding effectiveness of adhesives revealed that glass ionomers most effectively and durably bond to tooth tissue. Therefore, the result of this study that the adhesive strength of AIC was almost the same as that of GIC was considered to indicate that AIC has reliable adhesion [39]. 

In our previous studies, we demonstrated that porous spherical-shaped HAp improved not only mechanical strengths [19,20,21,22,23], but also the release properties of fluoride and the other ions [19,20,21,22,23] as well as the antibacterial property [19,21,23] of conventional GICs. In previous studies [19,20,21,22,23], the amount of GIC glass powder was relatively reduced to mix HAp particles with the GIC glasses in the AIC group. In other words, the total powder/liquid ratio (P/L) between the sample groups was unified in the GIC-1.2 and AIC-0.9 groups in the present study. However, fluoride is contained only in GIC glass, fluoroaluminosilicate glass, and not in HAp. Therefore, a question arose as to why the amount of fluoride releasing from AIC increased compared with that from GIC, despite the decrease in the amount of GIC glass powder containing fluoride. In Table 4, GIC-1.2 and AIC-0.9 are compared with the same total P/L. The amount of fluoride ions releasing from AIC-0.9 was significantly higher than that from GIC-1.2; this result was consistent with the results of the previous studies. Moreover, there were no significant differences in the fluoride ion release property for the GICs and AICs with the same Fuji III P/L. These results indicate that the fluoride ion releasing amounts correlate with the Fuji III P/L, that is, it is determined by the amounts of glass powder and polyacrylic acid solution, which eroded the glasses. Thus, it was suggested that porous spherical-shaped HAp particles may not have a direct impact on the modification of the fluoride ion release property. 

Solubility is also an important factor in assessing the quality of dental materials. Conventional GICs are particularly susceptible to moisture during the initial setting period, which can result in an increased solubility. In addition, since the solubility of cement could affect the elution of fluoride ions, we evaluated the solubility of AIC. In the present study, the solubility showed the same tendency as the fluoride ion release property of GIC and AIC in Table 4—there were no significant differences between the properties of GICs and AICs with the same Fuji III P/L. However, there was a significant difference in solubility between GIC-1.2 and AIC-0.9, which have the same total P/L. In addition, compared between GIC-0.9 and GIC-1.2 and between AIC-0.9 and AIC-1.2, the specimens with lower Fuji III P/L have higher solubility, respectively. This suggested that the solubility of AIC did not increase by the addition of HAp but by the correspondence of P/L. Moreover, we found that solubility is related to the elution amount of fluoride ions.

In the present study, the pH changes in small amounts of deionized water in contact with the surface of GIC and AIC specimens were determined using a flat pH electrode. The method of measuring surface pH in this study was modified from Ban et al.’s method [40,41]. The results in Figure 1 and Figure 2 show the changes in the surface pH of each specimen during setting and after long-term storage after setting, respectively. During the initial setting until 1 h after initiating mixing (Figure 1), the surface pH of GIC-1.2 was the highest, and it was significantly higher than those of AICs. However, after long-term storage (Figure 2), the surface pH values of AICs were greater than that of GIC-1.2 after 3 weeks. Therefore, we inferred that the surface pH of GICs increases immediately in the initial stage of setting; however, the surface pH of AICs increases gradually after setting and becomes higher than that of GICs. Several researchers mentioned that GICs could inhibit the in vitro growth of some oral bacterial species because of their low initial pH [42,43]. In our previous study [18,22], we reported that the antibacterial property of AICs against *Streptococcus mutans* incubated for 4 h was significantly greater than that of GICs for a pit and fissure sealant. We previously thought that the antibacterial property of AICs was mainly attributable to fluoride and the other ions’ release, but considering the result from short-time incubation, it might have been caused by the surface pH of specimens. Considering the results of a short incubation, the combined effect of the surface pH of the specimen and the sustained release of ions imbues the antibacterial properties.

The results in Figure 3 show the lactic-acid buffering capacity of GICs and AICs after setting. The acid buffer capacity of AIC groups when immersed in lactic acid for 30 min at 1 week after mixing was significantly higher than that in the GIC groups. However, the acid buffer capacity of AIC groups after 60 min was no different from that of GIC groups at the same time. This result may be clinically effective considering that the acid buffering ability is excellent for approximate 30 min during eating and drinking. The improvement in acid buffering is thought to be caused by the Ca ion elution from HAp. Identical to the results of surface pH measurement, the pH of lactic-acid-immersed GIC-1.2 specimens rapidly increased compared to those of immersed AIC specimens during setting; however, those of AICs rapidly increased compared to those of GICs after long-term storage after setting. Since the same tendency was observed in the surface pH measurement results, we considered that the increase in surface pH was involved in the acid buffering capacity of GICs and AICs. 

Although the results obtained in this study are expected to have effects on caries prevention and remineralization, no direct experiments have yet been conducted. It will become clear in near future research. In addition, regarding the mechanical strength, additional experiments such as repeated load experiments and thermal cycle tests were considered necessary. 

## 5. Conclusions

Porous spherical-shaped HAp particles change the matrix of GIC by participating in the reaction during the curing reaction, and they improve the mechanical and functional properties of conventional GIC. Therefore, we conclude that the novel AIC developed in this study is a clinically effective dental material for prevention and remineralization of tooth and initial carious lesion.

## Figures and Tables

**Table 1:** Compositions of the specimens.

Group	Powders			Fuji III Liquid (g)	Total P/L ^1^	Fuji III P/L ^1^
	Fuji III (g)	HApS (g)	Total (g)			
GIC-0.9	0.76	0	0.76	0.83	0.9	0.9
GIC-1.2	1.00	0	1.00	0.83	1.2	1.2
AIC-0.9	0.76	0.24	1.00	0.83	1.2	0.9
AIC-1.2	1.00	0.24	1.24	0.83	1.5	1.2

^1^ P/L, powder and liquid ratio.

**Table 2:** Results of flexural strength, compressive strength, and fracture toughness tests.

Group	Flexural Strength (MPa)		Compressive Strength (MPa)		Fracture Toughness (MPa·m^1/2^)	
	Mean (SD)		Mean (SD)		Mean (SD)	
GIC-0.9	7.7 (1.0)	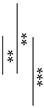	75.4 (8.2)	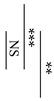	0.13 (0.02)	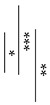
GIC-1.2	8.1 (1.3)		111.3 (2.1)		0.18 (0.04)	
AIC-0.9	15.6 (3.8)		117.2 (5.4)		0.22 (0.04)	
AIC-1.2	22.9 (2.9)		130.3 (8.0)		0.24 (0.03)	

*t*-test–NS, no significant difference; * *p* < 0.05, ** *p* < 0.01, *** *p* < 0.001.

**Table 3:** Results of shear bond strength to bovine enamel.

Group	Mean (SD) of Shear Bond Strength (MPa)					
	24 h		1 month		3 months	
GIC-0.9	2.4 (0.5)	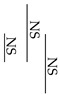	4.7 (0.9) ^†††^	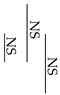	3.7 (0.9) ^††^	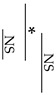
GIC-1.2	2.9 (0.8)		4.7 (0.6) ^†††^		4.1 (0.8) ^††^	
AIC-0.9	2.4 (0.7)		5.2 (2.0) ^††^		4.7 (1.0) ^†††^	
AIC-1.2	3.1 (0.6)		6.2 (2.1) ^††^		5.4 (1.9) ^††^	

*t*-test–* *p* < 0.05, NS no significant difference (comparison between groups with the same storage time). ^††^ *p* < 0.01, ^†††^ *p* < 0.001 (comparison to value for 24 h for the same group).

**Table 4:** Results of fluoride ion release and solubility.

Group	Cumulative Fluoride Ion Release Dose for 5 Days (µg/cm^2^)		Solubility for 24 h (%)	
	Mean (SD)		Mean (SD)	
GIC-0.9	379.9 (42.3)	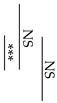	0.47 (0.03)	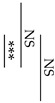
GIC-1.2	197.2 (23.8)		0.26 (0.04)	
AIC-0.9	382.8 (19.1)		0.44 (0.24)	
AIC-1.2	231.8 (24.4)		0.24 (0.03)	

*t*-test—NS, no significant difference; *** *p* < 0.001.
